# Combined Assessment of Serum dp-ucMGP and Albumin in Relation to All-Cause and Cardiovascular Mortality in Hemodialysis Patients

**DOI:** 10.3390/ijms27156596

**Published:** 2026-07-24

**Authors:** Vladana Stojiljkovic, Nikola Stefanovic, Jelena Basic, Branka Djordjevic, Jana Kocic, Branislav Apostolovic, Jelena Milenkovic, Vladan Cosic, Tatjana Cvetkovic

**Affiliations:** 1 Department of Biochemistry, Medical Faculty, University of Nis, Blvd. Zoran Djindjic 81, 18000 Nis, Serbia; jelena.basic@medfak.ni.ac.rs (J.B.); branka.djordjevic@medfak.ni.ac.rs (B.D.); jana.kocic@medfak.ni.ac.rs (J.K.); tatjana.cvetkovic@medfak.ni.ac.rs (T.C.); 2 Center for Medical and Clinical Biochemistry, University Clinical Center Nis, Blvd. Zoran Djindjic 48, 18000 Nis, Serbia; vl.cosic@gmail.com; 3 Department of Pharmacy, Medical Faculty, University of Nis, Blvd. Zoran Djindjic 81, 18000 Nis, Serbia; nikola.stefanovic@medfak.ni.ac.rs; 4 Clinic for Nephrology “Prof. Dr Spira Strahinjic”, University Clinical Center Nis, Blvd. Zoran Djindjic 48, 18000 Nis, Serbia; bane.apostolovic@gmail.com; 5 Department of Pathophysiology, Medical Faculty, University of Nis, Blvd. Zoran Djindjic 81, 18000 Nis, Serbia; jelena.milenkovic@medfak.ni.ac.rs

**Keywords:** dp-ucMGP, albumin, hemodialysis, all-cause mortality, cardiovascular mortality, chronic kidney disease

## Abstract

Dephosphorylated uncarboxylated matrix Gla protein (dp-ucMGP) is considered a marker of vitamin K status and vascular calcification risk in chronic kidney disease. This study assessed the association of serum dp-ucMGP, alone and combined with albumin, with all-cause and cardiovascular mortality in maintenance hemodialysis patients. This single-center observational cohort study included 133 maintenance hemodialysis patients with a two-year survival follow-up. Analyses involving dp-ucMGP and albumin were performed in 113 patients with complete biomarker data. Baseline serum dp-ucMGP, albumin, inflammatory markers, and routine laboratory parameters were measured. Associations with all-cause and cardiovascular mortality were evaluated using group comparisons, ROC curve analysis, Kaplan–Meier analysis, and Cox regression. Patients who died had significantly lower dp-ucMGP and albumin levels and higher inflammatory and hematological indices than survivors, with similar findings for cardiovascular mortality. The combined dp-ucMGP–albumin model showed higher apparent discriminatory performance than either marker alone for all-cause mortality (AUC 0.793, 95% CI 0.708–0.878, *p* < 0.001) and cardiovascular mortality (AUC 0.778, 95% CI 0.687–0.868, *p* < 0.001). Lower dp-ucMGP levels were associated with worse survival. In multivariable Cox regression, higher dp-ucMGP and albumin were independently associated with lower mortality. Combined dp-ucMGP and albumin assessment may provide complementary prognostic information in hemodialysis patients but requires validation in larger cohorts.

## 1. Introduction

Chronic kidney disease (CKD) is defined as structural or functional kidney impairment persisting for more than three months [1]. According to data from 2017 and 2021, CKD affects an estimated 9% of the global population, with 0.07% of individuals being in the final stage of the disease and 0.041% undergoing dialysis treatment [2,3]. The hospitalization rate among patients with CKD is 1.4 times higher than in the general population without kidney disease, with cardiovascular complications and bacterial infections being the most common causes [4]. Vascular calcification represents a pathological substrate present in approximately 90% of patients with end-stage CKD and significantly contributes to adverse cardiovascular events and mortality [5,6].

Matrix Gla protein (MGP) is an 84-amino-acid polypeptide synthesized in vascular smooth muscle cells and chondrocytes [7]. Its active form acts as an inhibitor of mineralization [8], whereas the inactive, dephosphorylated uncarboxylated form (dp-ucMGP) has been shown to predict the extent of vascular calcification in patients with CKD [9]. Circulating dp-ucMGP levels have also been associated with adverse outcomes, including mortality, in valvular aortic stenosis, heart failure, and peripheral artery disease [10].

Inflammation is a common risk factor for poor outcome in end-stage CKD [11]. Patients with end-stage CKD have been shown to have increased serum levels of inflammatory markers, including C-reactive protein (CRP), tumor necrosis factor alpha (TNF-alpha) and interleukin-6 (IL-6) [12]. Persistent inflammation may be associated with malnutrition in hemodialysis patients, as proinflammatory cytokines can contribute to anorexia, increased muscle protein breakdown, and muscle atrophy [13].

Assessment of malnutrition may represent an important component of follow-up in hemodialysis patients, since malnutrition has been identified as an independent risk factor for mortality [14,15]. Dietary restrictions commonly recommended to hemodialysis patients, together with increased protein requirements, may increase susceptibility to malnutrition [16]. As the most abundant serum protein, albumin is commonly used as an indicator of nutritional status. However, its interpretation remains debated, since serum albumin levels can be influenced by inflammation and other pathophysiological mechanisms, including excessive loss in nephrotic syndrome [17].

This study aimed to assess whether serum dp-ucMGP, both alone and in combination with albumin, is associated with all-cause and cardiovascular mortality in patients undergoing maintenance hemodialysis.

## 2. Results

### 2.1. Baseline Characteristics of the Study Cohort

The study cohort included 133 maintenance hemodialysis patients. The median age was 65.0 years (IQR 56.0–72.0), and the median dialysis vintage was 43.0 months (IQR 19.0–66.0). Most patients were male, 94/133 (70.7%). Hypertension was the most frequent comorbidity, present in 115 patients (86.5%), followed by diabetes mellitus in 28 patients (21.1%) and previous myocardial infarction in 17 patients (12.8%). The median serum albumin concentration was 37.0 g/L (IQR 35.0–39.0), while the median dp-ucMGP level was 1.46 ng/mL (IQR 1.19–1.80). The median CRP and IL-6 levels were 3.20 mg/L (IQR 1.70–8.60) and 11.30 pg/mL (IQR 6.27–22.90), respectively (Table 1).

### 2.2. Comparison According to All-Cause Mortality

During follow-up, 33 patients died, while 100 patients survived. Patients who died were significantly older than survivors (69.0 years (IQR 60.0–74.0) vs. 62.5 years (IQR 54.0–70.25), *p* = 0.019). They also tended to have lower dialysis adequacy, reflected by lower Kt/V values (1.16 ± 0.22 vs. 1.26 ± 0.28, *p* = 0.050).

Several inflammatory and hematological parameters differed significantly between survivors and non-survivors. Deceased patients had higher IL-6 levels (17.19 (IQR 8.22–38.80) vs. 9.72 (IQR 6.00–20.34), *p* = 0.015), higher neutrophil percentage (77.30% (IQR 75.20–80.10) vs. 73.20% (IQR 66.80–78.43), *p* = 0.008) and higher NLR (3.90 (IQR 3.44–4.90) vs. 3.12 (IQR 2.27–4.23), *p* = 0.004). Conversely, lymphocyte percentage was significantly lower in the deceased group (19.40% (IQR 15.90–22.00) vs. 23.25% (IQR 19.07–29.12), *p* = 0.006).

Markers of nutritional and metabolic status also differed between groups. Non-survivors had lower serum creatinine levels (845.67 ± 200.29 vs. 932.91 ± 234.03, *p* = 0.042), lower total protein levels (63.27 ± 6.03 vs. 65.77 ± 4.75, *p* = 0.035) and lower albumin levels (36.0 g/L (IQR 30.0–38.0) vs. 37.0 g/L (IQR 35.0–40.0), *p* = 0.008). Median dp-ucMGP levels were also significantly lower among non-survivors compared with survivors (1.34 ng/mL (IQR 1.11–1.46) vs. 1.51 ng/mL (IQR 1.27–1.97), *p* < 0.001).

Among categorical variables, previous cerebrovascular insult was more frequent in non-survivors than in survivors (5/33 (15.2%) vs. 2/100 (2.0%), *p* = 0.010), while no significant differences were observed for sex, smoking status, hypertension or diabetes mellitus (Table 2, full comparison is shown in Table A1).

### 2.3. Comparison According to Cardiovascular Mortality

Cardiovascular death occurred in 22 patients, while 111 patients did not experience cardiovascular death. Patients who died from cardiovascular causes were significantly older than those without cardiovascular death (69.5 years (IQR 65.0–73.75) vs. 62.0 years (IQR 54.0–71.0), *p* = 0.007).

The cardiovascular mortality group showed a more pronounced inflammatory profile. Interleukin-6 levels were significantly higher in patients with cardiovascular death (20.57 (IQR 9.57–36.07) vs. 9.85 (IQR 6.13–20.68), *p* = 0.022). These patients also had lower lymphocyte percentage (19.60% (IQR 15.83–21.77) vs. 22.90% (IQR 18.60–28.70), *p* = 0.031), lower absolute lymphocyte count (1.06 (IQR 0.93–1.48) vs. 1.40 (IQR 1.10–1.71), *p* = 0.007), higher neutrophil percentage (77.50% (IQR 75.20–80.70) vs. 73.70% (IQR 67.60–78.60), *p* = 0.033), and higher NLR (3.95 (IQR 3.53–4.92) vs. 3.17 (IQR 2.31–4.25), *p* = 0.016).

Patients with cardiovascular mortality had lower creatinine levels (808.00 ± 174.97 vs. 931.73 ± 232.99, *p* = 0.007) and lower albumin levels (36.0 g/L (IQR 30.0–38.0) vs. 37.0 g/L (IQR 35.0–40.0), *p* = 0.013). Median dp-ucMGP levels were also significantly lower in the cardiovascular mortality group (1.24 ng/mL (IQR 1.06–1.46) vs. 1.49 ng/mL (IQR 1.26–1.87), *p* = 0.004).

Smoking was more frequent among patients who experienced cardiovascular death (11/22 (50.0%) vs. 31/111 (27.9%), *p* = 0.042). Previous cerebrovascular insult was also significantly more prevalent in this group (4/22 (18.2%) vs. 3/111 (2.7%), *p* = 0.014) (Table 3, full comparison is shown in Table A2).

Spearman correlation analysis was performed to assess the relationship between dp-ucMGP and selected inflammatory and nutritional markers. Dephosphorylated-uncarboxylated MGP was not significantly correlated with albumin (ρ = 0.047, *p* = 0.618), CRP (ρ = 0.119, *p* = 0.208), IL-6 (ρ = 0.093, *p* = 0.328), NLR (ρ = −0.064, *p* = 0.499) or creatinine (ρ = 0.172, *p* = 0.069). In contrast, albumin showed significant inverse correlations with CRP, IL-6 and NLR (*p* < 0.001) (Table A3).

### 2.4. ROC Curve Analysis

**Figure 1:**
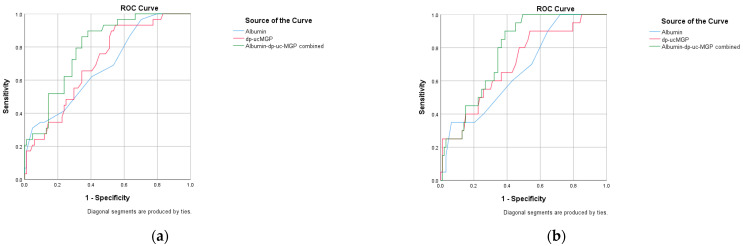
Receiver Operating Characteristic (ROC) curves of albumin, dp-ucMGP and the combined model in all-cause (**a**) and cardiovascular (**b**) mortality prediction.

Albumin data were available for all 133 patients, whereas dp-ucMGP measurements were unavailable in 20 patients; therefore, ROC, Kaplan–Meier, and Cox regression analyses were performed in 113 patients, including 29 all-cause deaths and 20 cardiovascular deaths. Included and excluded patients were generally similar with respect to demographic, clinical, laboratory, and mortality characteristics (Table A4). ROC curve analysis was performed to evaluate the discriminatory ability of dp-ucMGP, albumin, and their combined logistic regression model for all-cause and cardiovascular mortality. For all-cause mortality, the combined dp-ucMGP–albumin model showed the numerically highest apparent AUC of 0.793 (95% CI 0.708–0.878, *p* < 0.001) (Table 4, Figure 1).

Individually, dp-ucMGP demonstrated an AUC of 0.704 (95% CI 0.601–0.806, *p* = 0.001) for all-cause mortality. The optimal cutoff was ≤1.595 ng/mL. Albumin had an AUC of 0.686 (95% CI 0.576–0.795, *p* = 0.003).

For cardiovascular mortality, the combined dp-ucMGP–albumin model again showed the numerically highest AUC of 0.778 (95% CI 0.687–0.868, *p* < 0.001). Individually, dp-ucMGP had an AUC of 0.705 (95% CI 0.583–0.827, *p* = 0.004), while albumin had an AUC of 0.670 (95% CI 0.547–0.793, *p* = 0.017) (Table 4, Figure 1).

Bootstrap internal validation showed modest optimism in the combined models. For all-cause mortality, the apparent AUC was 0.793, the mean optimism was 0.009, and the optimism-corrected AUC was 0.784. For cardiovascular mortality, the corresponding values were 0.777, 0.017 and 0.760, respectively (Table A5).

### 2.5. Kaplan–Meier Survival Analysis According to dp-ucMGP Cutoff

**Figure 2:**
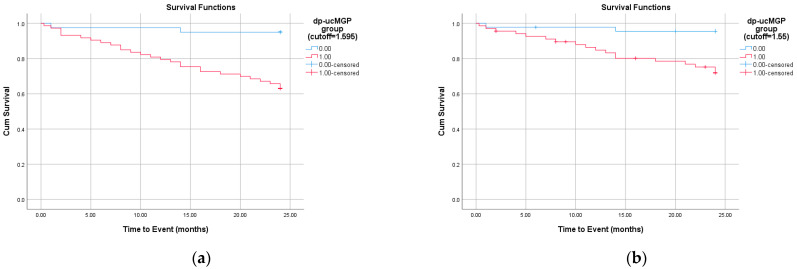
Kaplan–Meier curves of all-cause (**a**) and cardiovascular (**b**) mortality according to ROC-derived dp-ucMGP cutoff values. ‘dp-ucMGP’—dephosphorylated uncarboxylated Matrix Gla Protein.

Kaplan–Meier analysis was performed using the dp-ucMGP cutoff value derived from ROC analysis for all-cause mortality, namely 1.595 ng/mL. Patients with dp-ucMGP levels ≤ 1.595 ng/mL had significantly worse all-cause survival compared with those with levels > 1.595 ng/mL. In the higher dp-ucMGP group, only 2/40 patients experienced the event, while in the lower dp-ucMGP group, 27/73 patients died. Mean survival was 23.18 months (95% CI 21.973–24.377) in the >1.595 ng/mL group and 19.40 months (95% CI 17.636–21.159) in the ≤1.595 ng/mL group. The difference between survival curves was statistically significant by log-rank test (χ^2^ = 12.887, *p* < 0.001). Median survival was not reached in either group (Table 5, Figure 2).

For cardiovascular mortality, Kaplan–Meier analysis was performed using the cardiovascular mortality-specific ROC-derived dp-ucMGP cutoff of 1.55 ng/mL. This cutoff significantly stratified patients according to cardiovascular survival. Cardiovascular death occurred in 2/45 patients with dp-ucMGP > 1.55 ng/mL and in 18/68 patients with dp-ucMGP ≤ 1.55 ng/mL. Mean survival was 23.26 months (95% CI 22.187–24.336) in the higher dp-ucMGP group and 20.61 months (95% CI 18.939–22.289) in the lower dp-ucMGP group. The log-rank test confirmed a significant difference between groups (χ^2^ = 9.182, *p* = 0.002). Median survival was not reached in either group (Table 5, Figure 2).

### 2.6. Cox Proportional Hazards Regression Analysis

In the multivariable Cox regression model for all-cause mortality adjusted for albumin, dp-ucMGP, and age, dp-ucMGP remained independently associated with mortality. To improve interpretability, hazard ratios were rescaled to correspond to a 0.1 ng/mL increase in dp-ucMGP, a 5 g/L increase in albumin and a 10-year increase in age. Higher albumin levels were associated with a lower risk of death (HR 0.447, 95% CI 0.311–0.642, *p* < 0.001). Similarly, higher dp-ucMGP levels were independently associated with a lower risk of all-cause mortality (HR 0.846, 95% CI 0.768–0.931, *p* = 0.001) (Table 6). Separate extended Cox models including interactions between each covariate and ln(time/12) showed no evidence of time-varying effects for albumin, dp-ucMGP or age (Table A6).

For cardiovascular mortality, a parsimonious multivariable Cox regression model including dp-ucMGP, albumin, age, and smoking was used because of the limited number of cardiovascular events. Both dp-ucMGP and albumin remained associated with the outcome. Higher dp-ucMGP levels were associated with a lower risk of cardiovascular death (HR 0.870, 95% CI 0.779–0.971, *p* = 0.013), as were higher albumin levels (HR 0.440, 95% CI 0.272–0.712, *p* = 0.001). Older age (HR 1.665, 95% CI 1.020–2.718, *p* = 0.043) and smoking (HR 3.763, 95% CI 1.389–10.194, *p* = 0.009) were also associated with an increased risk of cardiovascular mortality (Table 6). Separate extended Cox models showed no evidence of time-varying effects for dp-ucMGP, albumin, age or smoking in the cardiovascular mortality model (Table A6).

At baseline, 11 patients (8.3%) were receiving oral vitamin K antagonist therapy, including 9 patients in the complete-case subgroup. After excluding these patients, 104 patients remained, including 27 all-cause deaths and 18 cardiovascular deaths. The associations of dp-ucMGP and albumin with both outcomes and the discriminatory performance of the combined model remained similar to those observed in the primary analyses (Table A7).

## 3. Discussion

In this cohort of maintenance hemodialysis patients, lower serum dp-ucMGP and albumin levels were associated with adverse survival outcomes, while their combined assessment showed higher apparent discriminatory performance compared with either marker alone. Previous studies have established the role of dp-ucMGP as a marker of vascular calcification and cardiovascular risk in CKD [18,19]. Based on this background, the present study evaluated the association of dp-ucMGP with all-cause and cardiovascular mortality in a hemodialysis cohort characterized by complex nutritional and inflammatory disturbances. In this context, albumin, CRP, IL-6 and complete blood count-derived inflammatory indices were assessed to provide a broader clinical framework for interpreting the relationship between dp-ucMGP and patient outcomes.

In the present study, patients with adverse outcomes showed a more pronounced inflammatory and catabolic profile. Non-survivors had higher IL-6 levels and higher NLR, while lymphocyte percentage was lower. Similar findings were observed in patients who died from cardiovascular causes, supporting the relevance of systemic inflammation in adverse outcomes among maintenance hemodialysis patients. Although CRP is widely used as an inflammatory marker, it is not entirely specific [20]. Interleukin-6 is involved in early inflammatory responses and stimulates hepatic CRP production [21] and has been investigated as an early marker of infection in clinical settings [22]. In CKD and dialysis populations, elevated IL-6 levels have also been described as part of the chronic inflammatory profile [23]. In our cohort, clinically apparent acute infections were excluded; therefore, the observed IL-6 elevation may more likely reflect persistent low-grade inflammation rather than acute infection. This inflammatory pattern, together with lower albumin levels, may indicate a combined inflammatory and catabolic state commonly observed in patients undergoing hemodialysis [24,25].

Beyond classical inflammatory markers, indices derived from the complete blood count (CBC) may provide additional insight into the inflammatory status of hemodialysis patients. The NLR has emerged as an accessible and cost-effective indicator of systemic inflammation and immune dysregulation, particularly in chronic diseases [26]. In the present study, higher NLR values were observed in patients with all-cause and cardiovascular mortality, reflecting the combined pattern of higher neutrophil predominance and lower lymphocyte-related parameters. Previous studies have associated NLR and platelet-to-lymphocyte ratio (PLR) with all-cause mortality in hemodialysis patients [27], while other composite inflammatory indices such as systemic immune-inflammation index (SII) have also been linked to prognosis in this population [28]. Unlike IL-6, which may reflect more dynamic cytokine activity [21], CBC-derived indices may capture more sustained changes in immune cell distribution, offering complementary insight into the chronic inflammatory milieu [29].

An important finding of the present study was the consistent association between lower serum dp-ucMGP levels and adverse outcomes. Patients who died during follow-up had significantly lower dp-ucMGP concentrations than survivors, and the same pattern was observed for cardiovascular mortality. This direction of association is noteworthy, as dp-ucMGP is generally considered an inactive form of MGP and a surrogate marker of poor vitamin K status and vascular calcification risk. It contrasts with findings from previous studies in diabetic CKD and population-based cohorts, in which higher circulating dp-ucMGP levels were associated with all-cause mortality and/or adverse cardiovascular outcomes [18,19]. Similar associations between higher dp-ucMGP levels and mortality or cardiovascular events have been reported in patients with chronic stable vascular disease and in patients undergoing continuous ambulatory peritoneal dialysis [30,31]. Earlier studies in end-stage renal disease (ESRD) and CKD have also linked inactive MGP forms with poorer survival or a greater vascular calcification burden [32,33], while more recent clinical cohorts likewise associated higher dp-ucMGP levels with worse outcomes [34,35]. Therefore, the lower dp-ucMGP concentrations observed among non-survivors in our cohort should be interpreted with caution and may reflect the specific clinical and metabolic context of maintenance hemodialysis patients. One possible explanation is that dp-ucMGP levels in this population may not reflect vascular calcification burden alone but may also be influenced by nutritional and inflammatory disturbances. In our cohort, lower dp-ucMGP levels were accompanied by lower albumin levels and a more pronounced inflammatory profile. Hypoalbuminemia in chronic diseases is not only a marker of nutritional depletion, but may also reflect inflammation, catabolism, and altered protein metabolism [36]. In hemodialysis patients, dp-ucMGP levels may also be affected by vitamin K status, supplementation, dialysis-related factors, and broader metabolic disturbances [37]. Thus, the unexpected direction of association observed in our study may indicate that dp-ucMGP should not be interpreted solely as an isolated marker of vascular calcification, but rather within the wider context of nutritional and inflammatory status.

The ROC analysis further supported the potential prognostic relevance of dp-ucMGP and albumin in mortality risk assessment. Although both markers showed significant discriminatory ability when analyzed separately, their combined model demonstrated higher AUC values for both all-cause and cardiovascular mortality. This suggests that dp-ucMGP and albumin may provide complementary information rather than reflecting identical risk pathways. The high negative predictive values observed for both outcomes also indicate that higher values of these markers were associated with a lower observed short-term mortality risk in this cohort, although the modest positive predictive values limit their use as standalone predictors of adverse outcomes.

Survival analysis confirmed that patients with lower dp-ucMGP levels had significantly worse outcomes. Using endpoint-specific ROC-derived cutoff values, Kaplan–Meier analysis showed lower survival in patients with dp-ucMGP levels ≤ 1.595 ng/mL for all-cause mortality and ≤1.55 ng/mL for cardiovascular mortality. These findings were supported by Cox regression analysis. In the all-cause mortality model, higher dp-ucMGP and albumin levels were independently associated with lower mortality after adjustment for age, and no significant time-varying effects were detected. In the exploratory cardiovascular mortality model, both markers remained independently associated with the outcome after adjustment for age and smoking, while older age and smoking were also associated with increased cardiovascular mortality. Given the limited number of cardiovascular events, this model should be interpreted as exploratory but provides supportive evidence for this association.

Albumin appears to be particularly important for interpreting these findings. As a widely used marker of nutritional and inflammatory status [38,39], serum albumin integrates several clinically relevant processes in hemodialysis patients, including protein-energy wasting, inflammation, catabolic burden and overall disease severity. The numerically higher AUC of the combined model suggests that these biomarkers may capture partly different aspects of risk. However, because the model was developed and evaluated in the same cohort, this finding should be regarded as exploratory. The lack of significant correlation between dp-ucMGP and albumin further suggests that these markers are not redundant. Rather than positioning dp-ucMGP as a standalone biomarker, these results support its potential role as part of a broader risk assessment framework in maintenance hemodialysis patients.

The absence of significant correlations between dp-ucMGP and albumin, CRP, IL-6, NLR or creatinine provides additional insight into the observed findings. Although patients with adverse outcomes exhibited both lower dp-ucMGP levels and a more pronounced low-protein, inflammatory–catabolic profile, dp-ucMGP was not directly associated with individual inflammatory or nutritional markers in correlation analysis. Therefore, its prognostic association may not be explained by a simple monotonic relationship with conventional markers of inflammation or nutritional status. Rather, lower dp-ucMGP may occur within a broader adverse clinical phenotype in which MGP metabolism, vitamin K status, protein turnover, supplementation, dialysis-related factors and unmeasured calcification burden may all contribute. Since vitamin K status, active MGP and direct measures of vascular calcification were not assessed, these findings should be interpreted primarily as clinical biomarker associations rather than evidence of a causal pathway.

This study has several limitations. First, it was conducted at a single center and included a relatively limited number of outcome events, particularly for cardiovascular mortality. For this reason, the cardiovascular Cox regression model should be considered exploratory. Although the combined dp-ucMGP–albumin model was internally validated using bootstrap resampling, it was developed and evaluated within the same cohort and lacked external validation; therefore, its discriminatory performance should still be considered preliminary. Second, dp-ucMGP measurements were unavailable in 20 patients, reducing the sample size for ROC, Kaplan–Meier, and Cox regression analyses to 113 patients. Although the included and excluded groups were generally similar across most examined characteristics, the use of a complete-case approach may still have influenced the resulting estimates. Third, data on vitamin K supplementation were incomplete. However, oral vitamin K antagonist use was identified, and exclusion of these patients did not materially alter the main findings. Finally, vitamin K status, active MGP, and direct vascular calcification measurements were not available, limiting mechanistic interpretation of the observed associations.

## 4. Materials and Methods

The study was conducted at the Nephrology Clinic “Prof. dr Spira Strahinjic”, University Clinical Center Nis, Serbia. It was designed as a single-center observational cohort study with a two-year survival follow-up. A total of 133 maintenance hemodialysis patients were enrolled in March 2023 and followed for two years. The primary outcomes were all-cause mortality and cardiovascular mortality. Cardiovascular mortality was defined as death attributed to documented cardiovascular events, myocardial infarction, stroke, heart failure, fatal arrhythmia, sudden cardiac death or other clearly documented cardiovascular causes, according to the available medical records. Baseline use of oral vitamin K antagonists was recorded from the patients’ medical records and was not an exclusion criterion. Data on vitamin K supplementation were incomplete and could not be reliably analyzed.

The inclusion criteria included thrice-weekly maintenance hemodialysis as regular renal replacement therapy, with patients treated with hemodialysis for more than three months continuously in the dialysis center of the Nephrology Clinic in University Clinical Center Nis, Serbia. Exclusion criteria included malignant comorbidities, clinically apparent acute infections, and autoimmune diseases. Clinical data were obtained from the electronic medical records, and blood samples were collected before the midweek dialysis session.

The study was approved by the Ethics Committees of the University Clinical Center Nis, Serbia (approval no. 35797/6, 1 December 2022) and the Faculty of Medicine, University of Nis, Serbia (approval no. 12-1285/2-3, 2 February 2023). It was conducted according to the Declaration of Helsinki and the principles of Good Clinical Practice and Good Laboratory Practice. All patients provided written informed consent prior to participation.

### 4.1. Sample Collection and Laboratory Analysis

Venous blood samples were collected into vacuum tubes containing potassium ethylenediaminetetraacetic acid [EDTA] as an anticoagulant. Complete blood count analysis was performed using a Celltac MEK-6510K automated hematology analyzer (Nihon Kohden, Tokyo, Japan). Samples were centrifuged at 2000× *g* for 10 min at room temperature using a Hettich Universal 320R centrifuge (Andreas Hettich GmbH, Tuttlingen, Germany). Plasma was separated and stored at −80 °C until further analysis.

Routine biochemical analyses were performed immediately from separate serum samples using standard methods in the Center for Medical and Clinical Biochemistry, University Clinical Center Nis, Serbia, on a Dimension RL automated analyzer (Siemens Healthineers, Brussels, Belgium). Interleukin-6 was measured using a Cobas e411 analyzer (Roche Diagnostics, Rotkreuz, Switzerland). Excess serum was stored at −80 °C until further analysis.

Serum dp-ucMGP concentration was measured using a commercially available ELISA kit (Cat. No EH4755, Fine Biotech, Wuhan, China), according to the manufacturer’s instructions. The dp-ucMGP levels were expressed in ng/mL, and the assay detection range was 0.078–5.000 ng/mL, with a sensitivity of 0.047 ng/mL. The mean intra-assay and inter-assay coefficients of variation were 5.27% and 4.68%, respectively.

### 4.2. Statistical Analysis

Statistical analyses, except for bootstrap internal validation, were performed using IBM SPSS Statistics for Windows, Version 20.0 (IBM Corp., Armonk, NY, USA). Bootstrap internal validation was performed in Python 3.13.5 (Python Software Foundation, Wilmington, DE, USA). Continuous variables were expressed as mean ± standard deviation or median and interquartile range, depending on data distribution, while categorical variables were presented as frequencies and percentages. The normality of distribution of continuous variables was assessed using the Kolmogorov–Smirnov test.

For group comparisons according to all-cause and cardiovascular mortality status, normally distributed continuous variables were analyzed using the independent-samples *t*-test or Welch’s *t*-test, as appropriate according to variance equality. Non-normally distributed continuous variables were compared using the Mann–Whitney U-test. Categorical variables were analyzed using the chi-square test or Fisher’s exact test, depending on expected cell frequencies. A *p* value < 0.05 was considered statistically significant.

Receiver operating characteristic (ROC) curve analysis was performed to evaluate the discriminatory ability of dp-ucMGP, albumin, and their combined model for all-cause and cardiovascular mortality. Area under the curve (AUC) values with 95% confidence intervals were reported. Optimal cutoff values were determined according to the Youden index. For the combined dp-ucMGP–albumin model, predicted probabilities obtained from logistic regression were used in ROC analysis. Patients with missing dp-ucMGP were excluded from ROC, Kaplan–Meier, and Cox regression analyses; therefore, these analyses were performed in 113 patients with complete data. To assess the potential for complete-case selection bias, baseline demographic, clinical, laboratory, and mortality characteristics were compared between patients included in the complete-case analyses and those excluded because of unavailable dp-ucMGP measurements. To account for potential optimism resulting from model development and evaluation in the same cohort, the combined dp-ucMGP–albumin logistic regression model was internally validated using 1000 bootstrap resamples. In each sample, the model was refitted and its AUC was calculated in both the bootstrap sample and the original cohort. The mean difference between these AUCs was subtracted from the apparent AUC to obtain the optimism-corrected AUC.

Survival analysis was performed using Kaplan–Meier curves and Cox proportional hazards regression models. For Kaplan–Meier analysis, patients were stratified according to the endpoint-specific ROC-derived dp-ucMGP cutoff values, and survival curves were compared using the log-rank test. Cox regression analysis was performed to evaluate the association of dp-ucMGP and albumin with all-cause and cardiovascular mortality. For clinical interpretability, hazard ratios for continuous variables were rescaled and presented per 0.1 ng/mL increase in dp-ucMGP, per 5 g/L increase in albumin, and per 10-year increase in age. The all-cause mortality model included dp-ucMGP, albumin and age. The cardiovascular mortality model included dp-ucMGP, albumin, age and smoking status, and was interpreted as exploratory due to the limited number of cardiovascular events. Choice of covariates included in the Cox regression analysis was predefined and clinically motivated to adjust the model to already known risk factors, present in the examined cohort. For cardiovascular mortality analyses, deaths from non-cardiovascular causes were treated as censored observations at the time of death. The proportional hazards assumption was assessed using separate extended Cox models that included an interaction between each covariate and log-transformed analysis time centered at 12 months. Each interaction was tested separately while retaining all main covariates of the corresponding primary model.

As a sensitivity analysis, the main Cox regression models and the combined dp-ucMGP–albumin ROC analyses were repeated after excluding patients receiving oral vitamin K antagonist therapy at baseline.

## 5. Conclusions

In conclusion, lower serum dp-ucMGP and albumin levels were associated with increased all-cause and cardiovascular mortality in maintenance hemodialysis patients. In this single-center cohort, their combined assessment showed higher apparent discriminatory performance than either marker alone, suggesting that dp-ucMGP may provide additional, non-redundant prognostic information when interpreted alongside albumin and markers of nutritional and inflammatory status. These findings require validation in larger independent cohorts.

## Figures and Tables

**Table 1:** Descriptive characteristics of the examined cohort.

Variable	Overall Mean ± SD or Overall Median (IQR) or Number of Patinets (N)/%
Age (years)	65.00 (56.00–72.00)
Sex (males)	94/133 (70.7%)
Smoking	42/133 (31.6%)
Hypertension	115/133 (86.5%)
Diabetes mellitus	28/133 (21.1%)
Myocardial infarction	17/133 (12.8%)
Cerebrovascular insult	7/133 (5.3%)
Coronary disease	5/133 (3.8%)
Heart failure	11/133 (8.3%)
Arrhythmia	7/133 (5.3%)
Oral vitamin K antagonist therapy	11/133 (8.3%)
Dialysis vintage (months)	43.00 (19.00–66.00)
Kt/V	1.23 ± 0.27
IL-6 (pg/mL)	11.30 (6.27–22.90)
WBC (×10^9^/L)	6.30 (5.20–7.50)
HGB (g/L)	104.23 ± 13.56
HCT (L/L)	0.31 ± 0.04
PLT (×10^9^/L)	206.00 (165.00–243.00)
LY%	22.00 (18.20–28.00)
MO%	3.40 (2.80–4.00)
NE%	74.70 (67.90–79.00)
LY# (×10^9^/L)	1.40 (1.02–1.70)
MO# (×10^9^/L)	0.20 (0.20–0.30)
NE# (×10^9^/L)	4.60 (3.70–5.75)
RDW (%)	14.30 (13.50–15.10)
MPV (fL)	6.30 ± 0.93
PDW (%)	17.49 ± 0.74
PCT (%)	0.12 (0.10–0.15)
NLR	3.40 (2.37–4.29)
Glucose (mmol/L)	5.50 (4.90–6.70)
Creatinine (µmol/L)	911.27 ± 228.55
Na (mmol/L)	137.00 (135.00–139.00)
K (mmol/L)	5.33 ± 0.89
Ca (mmol/L)	2.06 ± 0.19
P (mmol/L)	1.60 (1.30–1.95)
Cholesterol (mmol/L)	4.29 ± 1.02
Triglycerides (mmol/L)	1.31 (1.00–2.13)
Proteins (g/L)	65.15 ± 5.19
Albumin (g/L)	37.00 (35.00–39.00)
Uric acid (µmol/L)	362.00 (321.00–414.00)
Fe (µmol/L)	8.40 (6.40–10.60)
TIBC (µmol/L)	36.00 (31.00–41.00)
UIBC (µmol/L)	26.40 (21.70–31.90)
AST (U/L)	19.00 (16.00–25.00)
ALT (U/L)	16.00 (13.00–20.00)
ALP (U/L)	76.00 (61.00–95.00)
GGT (U/L)	24.00 (18.00–35.00)
CRP (mg/L)	3.20 (1.70–8.60)
dp-ucMGP (ng/mL)	1.46 (1.19–1.80)

‘Kt/V’—clearance × time/volume (dialysis efficacy indicator), ‘IL-6’—interleukin 6, ‘WBC’—white blood cells, ‘HGB’—hemoglobin, ‘HCT’—hematocrit, ‘PLT’—platelets, ‘LY%’—relative lymphocyte count, ‘MO%’—relative monocyte count, ‘NE%’—relative neutrophil count, ‘LY#’—absolute lymphocyte count, ‘MO#’—absolute monocyte count, ‘NE#’—absolute neutrophil count, ‘RDW’—red blood cells distribution width, ‘MPV’—mean platelets volume, ‘PDW’—platelets distribution width, ‘PCT’—plateletocrit, ‘NLR’—neutrophil–lymphocyte ratio, ‘Na’—sodium concentration in serum, ‘K’—potassium concentration in serum, ‘Ca’—calcium concentration in serum, ‘P’—phosphate concentration, ‘Fe’—iron concentration in serum, ‘TIBC’—total iron binding capacity, ‘UIBC’—unsaturated iron binding capacity, ‘AST’—aspartate aminotransferase, ‘ALT’—alanine aminotransferase, ’ALP’—alkaline phosphatase, ‘GGT’—gamma glutamyl transferase, ‘CRP’—C-reactive protein, ‘dp-ucMGP’—dephosphorylated uncarboxylated Matrix Gla Protein.

**Table 2:** Selected baseline characteristics according to all-cause mortality.

Variable	Survivors/Non-Event Mean ± SD or Median (IQR) or N/%	Deceased/Event Mean ± SD or Median (IQR) or N/%	Test	Statistic	*p*
Age (years)	62.50 (54.00–70.25)	69.00 (60.00–74.00)	Mann–Whitney U	1198.5	0.019
Sex (males)	68/100 (68.0%)	26/33 (78.8%)	Chi-square	1.393	0.238
Smoking	28/100 (28.0%)	14/33 (42.4%)	Chi-square	2.389	0.122
Hypertension	84/100 (84.0%)	31/33 (93.9%)	Fisher exact	/	0.239
Diabetes mellitus	20/100 (20.0%)	8/33 (24.2%)	Chi-square	0.269	0.604
Myocardial infarction	12/100 (12.0%)	5/33 (15.2%)	Chi-square	0.221	0.638
Cerebrovascular insult	2/100 (2.0%)	5/33 (15.2%)	Fisher exact	/	0.010
Coronary disease	3/100 (3.0%)	2/33 (6.1%)	Fisher exact	/	0.597
Heart failure	8/100 (8.0%)	3/33 (9.1%)	Fisher exact	/	1.000
Arrhythmia	5/100 (5.0%)	2/33 (6.1%)	Fisher exact	/	1.000
Dialysis vintage (months)	46.50 (20.75–67.00)	26.00 (15.00–61.00)	Mann–Whitney U	1863.5	0.267
Kt/V	1.26 ± 0.28	1.16 ± 0.22	Welch t	1.997	0.050
IL-6 (pg/mL)	9.72 (6.00–20.34)	17.19 (8.22–38.80)	Mann–Whitney U	1181	0.015
LY%	23.25 (19.07–29.12)	19.40 (15.90–22.00)	Mann–Whitney U	2180	0.006
NE%	73.20 (66.80–78.43)	77.30 (75.20–80.10)	Mann–Whitney U	1143.5	0.008
RDW (%)	14.05 (13.50–15.00)	14.90 (14.30–15.80)	Mann–Whitney U	1031	0.001
NLR	3.12 (2.27–4.23)	3.90 (3.44–4.90)	Mann–Whitney U	1091	0.004
Creatinine (µmol/L)	932.91 ± 234.03	845.67 ± 200.29	Welch t	2.078	0.041
Proteins (g/L)	65.77 ± 4.75	63.27 ± 6.03	Welch t	2.168	0.035
Albumin (g/L)	37.00 (35.00–40.00)	36.00 (30.00–38.00)	Mann–Whitney U	2154	0.008
Fe (µmol/L)	9.10 (6.92–10.72)	7.10 (5.30–10.00)	Mann–Whitney U	2068.5	0.029
GGT (U/L)	23.00 (17.00–30.25)	30.00 (21.00–49.00)	Mann–Whitney U	1098	0.004
CRP (mg/L)	3.05 (1.12–8.00)	4.30 (2.20–13.90)	Mann–Whitney U	1313.5	0.080
dp-ucMGP (ng/mL)	1.51 (1.27–1.97)	1.34 (1.11–1.46)	Mann–Whitney U	1719.5	<0.001

Data are expressed as mean ± standard deviation, median and interquartile range or number of the participants and percentage, depending on the type and distribution of data. ‘Kt/V’—clearance × time/volume (dialysis efficacy indicator), ‘IL-6’—interleukin 6, ‘LY%’—relative lymphocyte count, ‘NE%’—relative neutrophil count, ‘RDW’—red blood cells distribution width, ‘NLR’—neutrophil–lymphocyte ratio, ‘Fe’—iron concentration in serum, ‘GGT’—gamma glutamyl transferase, ‘CRP’—C-reactive protein, ‘dp-ucMGP’—dephosphorylated uncarboxylated Matrix Gla Protein. /: Fisher’s exact test does not return a specific statistic value.

**Table 3:** Selected baseline characteristics according to cardiovascular mortality.

Variable	Survivors/Non-Event Mean ± SD or Median (IQR) or N/%	Deceased/Event Mean ± SD or Median (IQR) or N/%	Test	Statistic	*p*
Age (years)	62.00 (54.00–71.00)	69.50 (65.00–73.75)	Mann–Whitney U	773	0.007
Sex (males)	77/111 (69.4%)	17/22 (77.3%)	Chi-square	0.553	0.457
Smoking	31/111 (27.9%)	11/22 (50.0%)	Chi-square	4.140	0.042
Hypertension	94/111 (84.7%)	21/22 (95.5%)	Fisher exact	/	0.305
Diabetes mellitus	22/111 (19.8%)	6/22 (27.3%)	Chi-square	0.614	0.433
Myocardial infarction	13/111 (11.7%)	4/22 (18.2%)	Fisher exact	/	0.482
Cerebrovascular insult	3/111 (2.7%)	4/22 (18.2%)	Fisher exact	/	0.014
Coronary disease	4/111 (3.6%)	1/22 (4.5%)	Fisher exact	/	1.000
Heart failure	8/111 (7.2%)	3/22 (13.6%)	Fisher exact	/	0.390
Arrhythmia	5/111 (4.5%)	2/22 (9.1%)	Fisher exact	/	0.327
Dialysis vintage (months)	45.00 (19.50–66.50)	28.00 (15.00–61.75)	Mann–Whitney U	1316	0.567
Kt/V	1.25 ± 0.28	1.17 ± 0.22	Welch t	1.366	0.180
IL-6 (pg/mL)	9.85 (6.13–20.68)	20.57 (9.57–36.07)	Mann–Whitney U	841	0.022
LY%	22.90 (18.60–28.70)	19.60 (15.83–21.77)	Mann–Whitney U	1578	0.031
NE%	73.70 (67.60–78.60)	77.50 (75.20–80.70)	Mann–Whitney U	868.5	0.033
LY# (×10^9^/L)	1.40 (1.10–1.71)	1.06 (0.93–1.48)	Mann–Whitney U	1669	0.006
RDW (%)	14.10 (13.50–15.00)	15.00 (14.33–15.73)	Mann–Whitney U	736.5	0.003
NLR	3.17 (2.31–4.25)	3.95 (3.53–4.92)	Mann–Whitney U	824	0.016
Creatinine (µmol/L)	931.73 ± 232.99	808.00 ± 174.97	Welch t	2.853	0.007
Triglycerides (mmol/L)	1.40 (1.03–2.23)	1.02 (0.93–1.56)	Mann–Whitney U	1560	0.040
Albumin (g/L)	37.00 (35.00–40.00)	36.00 (30.00–38.00)	Mann–Whitney U	1629	0.013
CRP (mg/L)	3.20 (1.40–8.20)	4.85 (2.23–13.75)	Mann–Whitney U	1022	0.229
dp-ucMGP (ng/mL)	1.49 (1.26–1.87)	1.24 (1.06–1.46)	Mann–Whitney U	1314.5	0.004

Data are expressed as mean ± standard deviation, median and interquartile range or number of the participants and percentage, depending on the type and distribution of data. ‘Kt/V’—clearance × time/volume (dialysis efficacy indicator), ‘IL-6’—interleukin 6, ‘LY%’—relative lymphocyte count, ‘NE%’—relative neutrophil count, ‘LY#’—absolute lymphocyte count, ‘RDW’—red blood cells distribution width, ‘NLR’—neutrophil–lymphocyte ratio, ‘CRP’—C-reactive protein, ‘dp-ucMGP’—dephosphorylated uncarboxylated Matrix Gla Protein. /: Fisher’s exact test does not return a specific statistic value.

**Table 4:** ROC analysis of dp-ucMGP, albumin, and the combined model.

Endpoint	Marker/Model	AUC	95% CI	*p* Value	Cutoff	Sensitivity	Specificity	Youden Index	PPV	NPV
All-cause mortality	Combined dp-ucMGP–albumin model	0.793	0.708–0.878	<0.001	Predicted probability ≥ 0.235	0.862	0.655	0.517	0.463	0.932
	Albumin	0.686	0.576–0.795	0.003	Albumin ≤ 39.5 g/L	0.966	0.298	0.263	0.322	0.962
	dp-ucMGP	0.704	0.601–0.806	0.001	dp-ucMGP ≤ 1.595 ng/mL	0.931	0.452	0.383	0.370	0.950
Cardiovascular mortality	Combined dp-ucMGP–albumin model	0.778	0.687–0.868	<0.001	Predicted probability ≥ 0.152	0.900	0.613	0.513	0.333	0.966
	Albumin	0.670	0.547–0.793	0.017	Albumin ≤ 31.0 g/L	0.350	0.935	0.285	0.538	0.870
	dp-ucMGP	0.705	0.583–0.827	0.004	dp-ucMGP ≤ 1.55 ng/mL	0.900	0.462	0.362	0.265	0.956

‘AUC’—area under the curve, ‘CI’—confidence interval, ‘PPV’—positive predictive value, ‘NPV’—negative predictive value, ‘dp-ucMGP’—dephosphorylated uncarboxylated Matrix Gla Protein.

**Table 5:** Kaplan–Meier survival analysis according to dp-ucMGP cutoff.

Endpoint	dp-ucMGP Group	N	Events	Censored N (%)	Mean Survival (Months)	95% CI	Log-Rank χ^2^	df	*p* Value
All-cause mortality	>1.595 ng/mL	40	2	38 (95.0%)	23.18	21.973–24.377	12.887	1	<0.001
	≤1.595 ng/mL	73	27	46 (63.0%)	19.40	17.636–21.159	12.887	1	<0.001
Cardiovascular mortality	>1.55 ng/mL	45	2	43 (95.6%)	23.26	22.187–24.336	9.182	1	0.002
	≤1.55 ng/mL	68	18	50 (73.5%)	20.61	18.939–22.289	9.182	1	0.002

Median survival was not determined as neither of the groups reached 50% event threshold required for median survival estimation. ‘dp-ucMGP’—dephosphorylated uncarboxylated Matrix Gla Protein, ‘CI’—confidence interval.

**Table 6:** Cox proportional hazards regression models.

Endpoint	Model	N	Events	Variable	B	SE	Wald	HR	95% CI	*p* Value
All-cause mortality	albumin + dp-ucMGP + age	113	29	Albumin, per 5 g/L	−0.805	0.185	18.386	0.447	0.311–0.642	<0.001
				dp-ucMGP, per 0.1 ng/mL	−0.168	0.049	11.705	0.846	0.768–0.931	0.001
				Age, per 10 years	0.050	0.170	0.094	1.051	0.753–1.467	0.759
Cardiovascular mortality	dp-ucMGP + albumin + age + smoking	113	20	dp-ucMGP, per 0.1 ng/mL	−0.140	0.056	6.162	0.870	0.779–0.971	0.013
				Albumin, per 5 g/L	−0.820	0.245	11.122	0.440	0.272–0.712	0.001
				Age, per 10 years	0.510	0.250	4.100	1.665	1.020–2.718	0.043
				Smoking, yes vs. no	1.325	0.508	6.795	3.763	1.389–10.194	0.009

Hazard ratios for continuous variables were rescaled for clinical interpretability and are presented per 5 g/L increase in albumin, per 0.1 ng/mL increase in dp-ucMGP, and per 10-year increase in age. Smoking is presented as yes versus no. ‘B’—regression coefficient, ‘SE’—standard error, ‘HR’—hazard ratio, ‘CI’—confidence interval.

## Data Availability

The data are not publicly available due to privacy and ethical restrictions but are available upon reasonable request from the corresponding author.

## Abbreviations

The following abbreviations are used in this manuscript:

CKD	Chronic Kidney Disease
MGP	Matrix Gla Protein
dp-ucMGP	dephosphorylated uncarboxylated Matrix Gla Protein
CRP	C-reactive protein
TNF-a	Tumor necrosis factor alpha
IL-6	Interleukin-6
EDTA	potassium ethylenediaminetetra-acetic acid
ROC	Receiver operating characteristic curve
AUC	Area under the curve
Kt/V	Urea clearance × time/volume
CBC	Complete blood count
NE	Neutrophil count
LY	Lymphocyte count
MO	Monocyte count
NLR	Neutrophil–lymphocyte ratio

## Appendix A

**Table A1:** Full baseline characteristics according to all-cause mortality.

Variable	Survivors/Non-Event Mean ± SD or Median (IQR) or N/%	Deceased/Event Mean ± SD or Median (IQR) or N/%	Test	Statistic	*p*
Age (years)	62.50 (54.00–70.25)	69.00 (60.00–74.00)	Mann–Whitney U	1198.5	0.019
Sex (males)	68/100 (68.0%)	26/33 (78.8%)	Chi-square	1.393	0.238
Smoking	28/100 (28.0%)	14/33 (42.4%)	Chi-square	2.389	0.122
Hypertension	84/100 (84.0%)	31/33 (93.9%)	Fisher exact	/	0.239
Diabetes mellitus	20/100 (20.0%)	8/33 (24.2%)	Chi-square	0.269	0.604
Myocardial infarction	12/100 (12.0%)	5/33 (15.2%)	Chi-square	0.221	0.638
Cerebrovascular insult	2/100 (2.0%)	5/33 (15.2%)	Fisher exact	/	0.010
Coronary disease	3/100 (3.0%)	2/33 (6.1%)	Fisher exact	/	0.597
Heart failure	8/100 (8.0%)	3/33 (9.1%)	Fisher exact	/	1.000
Arrhythmia	5/100 (5.0%)	2/33 (6.1%)	Fisher exact	/	1.000
Dialysis vintage (months)	46.50 (20.75–67.00)	26.00 (15.00–61.00)	Mann–Whitney U	1863.5	0.267
Kt/V	1.26 ± 0.28	1.16 ± 0.22	Welch t	1.997	0.050
IL-6 (pg/mL)	9.72 (6.00–20.34)	17.19 (8.22–38.80)	Mann–Whitney U	1181	0.015
WBC (×10^9^/L)	6.40 (5.20–7.20)	6.30 (5.10–8.00)	Mann–Whitney U	1583	0.729
HGB (g/L)	105.05 ± 13.13	101.76 ± 14.75	Welch t	1.141	0.259
HCT (L/L)	0.32 ± 0.04	0.31 ± 0.05	Welch t	0.822	0.415
PLT (×10^9^/L)	204.00 (165.00–243.25)	208.00 (161.00–243.00)	Mann–Whitney U	1644.5	0.979
LY%	23.25 (19.07–29.12)	19.40 (15.90–22.00)	Mann–Whitney U	2180	0.006
MO%	3.40 (2.80–4.10)	3.30 (2.90–3.80)	Mann–Whitney U	1818	0.382
NE%	73.20 (66.80–78.43)	77.30 (75.20–80.10)	Mann–Whitney U	1143.5	0.008
LY# (×10^9^/L)	1.40 (1.10–1.71)	1.10 (1.00–1.60)	Mann–Whitney U	2013	0.058
MO# (×10^9^/L)	0.20 (0.20–0.30)	0.20 (0.20–0.30)	Mann–Whitney U	1634.5	0.929
NE# (×10^9^/L)	4.30 (3.68–5.70)	4.90 (3.90–6.10)	Mann–Whitney U	1386.5	0.170
RDW (%)	14.05 (13.50–15.00)	14.90 (14.30–15.80)	Mann–Whitney U	1031	0.001
MPV (fL)	6.31 ± 0.91	6.28 ± 0.99	Welch t	0.130	0.897
PDW (%)	17.48 ± 0.77	17.53 ± 0.68	Welch t	−0.318	0.751
PCT (%)	0.12 (0.10–0.15)	0.13 (0.09–0.15)	Mann–Whitney U	1722.5	0.707
NLR	3.12 (2.27–4.23)	3.90 (3.44–4.90)	Mann–Whitney U	1091	0.004
Glucose (mmol/L)	5.50 (4.88–6.55)	5.70 (5.00–6.90)	Mann–Whitney U	1605.5	0.819
Creatinine (µmol/L)	932.91 ± 234.03	845.67 ± 200.29	Welch t	2.078	0.041
Na (mmol/L)	137.00 (135.00–139.00)	137.00 (134.00–139.00)	Mann–Whitney U	1725	0.696
K (mmol/L)	5.41 ± 0.83	5.09 ± 1.05	Welch t	1.607	0.115
Ca (mmol/L)	2.06 ± 0.17	2.04 ± 0.22	Welch t	0.615	0.541
P (mmol/L)	1.58 (1.30–1.93)	1.65 (1.40–1.95)	Mann–Whitney U	1568	0.671
Cholesterol (mmol/L)	4.35 ± 0.91	4.13 ± 1.30	Welch t	0.865	0.392
Triglycerides (mmol/L)	1.48 (1.03–2.29)	1.13 (0.92–1.61)	Mann–Whitney U	1983.5	0.083
Proteins (g/L)	65.77 ± 4.75	63.27 ± 6.03	Welch t	2.168	0.035
Albumin (g/L)	37.00 (35.00–40.00)	36.00 (30.00–38.00)	Mann–Whitney U	2154	0.008
Uric acid (µmol/L)	362.50 (324.50–406.00)	355.00 (319.00–420.00)	Mann–Whitney U	1631.5	0.925
Fe (µmol/L)	9.10 (6.92–10.72)	7.10 (5.30–10.00)	Mann–Whitney U	2068.5	0.029
TIBC (µmol/L)	36.00 (31.00–39.00)	38.00 (27.00–48.00)	Mann–Whitney U	1506	0.454
UIBC (µmol/L)	26.10 (22.03–30.25)	29.90 (21.50–37.10)	Mann–Whitney U	1410.5	0.213
AST (U/L)	19.00 (15.75–25.00)	19.00 (16.00–25.00)	Mann–Whitney U	1587.5	0.746
ALT (U/L)	16.00 (13.00–19.25)	17.00 (14.00–22.00)	Mann–Whitney U	1426	0.243
ALP (U/L)	79.00 (58.00–95.50)	70.00 (64.00–92.00)	Mann–Whitney U	1784.5	0.485
GGT (U/L)	23.00 (17.00–30.25)	30.00 (21.00–49.00)	Mann–Whitney U	1098	0.004
CRP (mg/L)	3.05 (1.12–8.00)	4.30 (2.20–13.90)	Mann–Whitney U	1313.5	0.080
dp-ucMGP (ng/mL)	1.51 (1.27–1.97)	1.34 (1.11–1.46)	Mann–Whitney U	1719.5	<0.001

Data are expressed as mean ± standard deviation, median and interquartile range or number of the participants and percentage, depending on the type and distribution of data. ‘Kt/V’—clearance × time/volume (dialysis efficacy indicator), ‘IL-6’—interleukin 6, ‘WBC’—white blood cells, ‘HGB’—hemoglobin, ‘HCT’—hematocrit, ‘PLT’—platelets, ‘LY%’—relative lymphocyte count, ‘MO%’—relative monocyte count, ‘NE%’—relative neutrophil count, ‘LY#’—absolute lymphocyte count, ‘MO#’—absolute monocyte count, ‘NE#’—absolute neutrophil count, ‘RDW’—red blood cells distribution width, ‘MPV’—mean platelets volume, ‘PDW’—platelets distribution width, ‘PCT’—plateletocrit, ‘NLR’—neutrophil–lymphocyte ratio, ‘Na’—sodium concentration in serum, ‘K’—potassium concentration in serum, ‘Ca’—calcium concentration in serum, ‘P’—phosphate concentration, ‘Fe’—iron concentration in serum, ‘TIBC’—total iron binding capacity, ‘UIBC’—unsaturated iron binding capacity, ‘AST’—aspartate aminotransferase, ‘ALT’—alanine aminotransferase, ’ALP’—alkaline phosphatase, ‘GGT’—gamma glutamyl transferase, ‘CRP’—C-reactive protein, ‘dp-ucMGP’—dephosphorylated uncarboxylated Matrix Gla Protein. /: Fisher’s exact test does not return a specific statistic value.

**Table A2:** Full baseline characteristics according to cardiovascular mortality.

Variable	Survivors/Non-Event Mean ± SD or Median (IQR) or N/%	Deceased/Event Mean ± SD or Median (IQR) or N/%	Test	Statistic	*p*
Age (years)	62.00 (54.00–71.00)	69.50 (65.00–73.75)	Mann–Whitney U	773	0.007
Sex (males)	77/111 (69.4%)	17/22 (77.3%)	Chi-square	0.553	0.457
Smoking	31/111 (27.9%)	11/22 (50.0%)	Chi-square	4.140	0.042
Hypertension	94/111 (84.7%)	21/22 (95.5%)	Fisher exact	/	0.305
Diabetes mellitus	22/111 (19.8%)	6/22 (27.3%)	Chi-square	0.614	0.433
Myocardial infarction	13/111 (11.7%)	4/22 (18.2%)	Fisher exact	/	0.482
Cerebrovascular insult	3/111 (2.7%)	4/22 (18.2%)	Fisher exact	/	0.014
Coronary disease	4/111 (3.6%)	1/22 (4.5%)	Fisher exact	/	1.000
Heart failure	8/111 (7.2%)	3/22 (13.6%)	Fisher exact	/	0.390
Arrhythmia	5/111 (4.5%)	2/22 (9.1%)	Fisher exact	/	0.327
Dialysis vintage (months)	45.00 (19.50–66.50)	28.00 (15.00–61.75)	Mann–Whitney U	1316	0.567
Kt/V	1.25 ± 0.28	1.17 ± 0.22	Welch t	1.366	0.180
IL-6 (pg/mL)	9.85 (6.13–20.68)	20.57 (9.57–36.07)	Mann–Whitney U	841	0.022
WBC (×10^9^/L)	6.40 (5.20–7.55)	6.05 (5.10–7.35)	Mann–Whitney U	1398.5	0.284
HGB (g/L)	104.73 ± 12.94	101.73 ± 16.46	Welch t	0.807	0.427
HCT (L/L)	0.31 ± 0.04	0.31 ± 0.05	Welch t	0.585	0.563
PLT (×10^9^/L)	206.00 (165.00–243.50)	200.50 (150.00–238.25)	Mann–Whitney U	1288	0.687
LY%	22.90 (18.60–28.70)	19.60 (15.83–21.77)	Mann–Whitney U	1578	0.031
MO%	3.40 (2.80–4.10)	3.15 (2.82–3.90)	Mann–Whitney U	1345.5	0.452
NE%	73.70 (67.60–78.60)	77.50 (75.20–80.70)	Mann–Whitney U	868.5	0.033
LY# (×10^9^/L)	1.40 (1.10–1.71)	1.06 (0.93–1.48)	Mann–Whitney U	1669	0.006
MO# (×10^9^/L)	0.20 (0.20–0.30)	0.20 (0.20–0.20)	Mann–Whitney U	1394.5	0.236
NE# (×10^9^/L)	4.30 (3.75–5.85)	4.90 (3.67–5.42)	Mann–Whitney U	1198	0.891
RDW (%)	14.10 (13.50–15.00)	15.00 (14.33–15.73)	Mann–Whitney U	736.5	0.003
MPV (fL)	6.30 ± 0.92	6.32 ± 0.99	Welch t	−0.089	0.929
PDW (%)	17.45 ± 0.78	17.72 ± 0.50	Welch t	−2.114	0.040
PCT (%)	0.12 (0.10–0.16)	0.13 (0.09–0.15)	Mann–Whitney U	1319	0.553
NLR	3.17 (2.31–4.25)	3.95 (3.53–4.92)	Mann–Whitney U	824	0.016
Glucose (mmol/L)	5.50 (4.90–6.45)	5.90 (5.12–7.05)	Mann–Whitney U	1055.5	0.317
Creatinine (µmol/L)	931.73 ± 232.99	808.00 ± 174.97	Welch t	2.853	0.007
Na (mmol/L)	137.00 (135.00–139.00)	137.00 (134.00–139.00)	Mann–Whitney U	1326.5	0.523
K (mmol/L)	5.37 ± 0.86	5.13 ± 1.03	Welch t	1.051	0.303
Ca (mmol/L)	2.06 ± 0.18	2.04 ± 0.22	Welch t	0.368	0.716
P (mmol/L)	1.58 (1.33–1.95)	1.65 (1.27–1.85)	Mann–Whitney U	1230.5	0.956
Cholesterol (mmol/L)	4.33 ± 0.97	4.10 ± 1.26	Welch t	0.822	0.418
Triglycerides (mmol/L)	1.40 (1.03–2.23)	1.02 (0.93–1.56)	Mann–Whitney U	1560	0.040
Proteins (g/L)	66.00 (62.00–69.50)	63.50 (60.25–67.00)	Mann–Whitney U	1509	0.081
Albumin (g/L)	37.00 (35.00–40.00)	36.00 (30.00–38.00)	Mann–Whitney U	1629	0.013
Uric acid (µmol/L)	364.00 (322.00–414.00)	346.50 (320.25–410.50)	Mann–Whitney U	1290	0.678
Fe (µmol/L)	8.80 (6.65–10.50)	7.30 (5.22–11.38)	Mann–Whitney U	1360	0.402
TIBC (µmol/L)	36.00 (31.00–39.50)	39.00 (27.00–49.00)	Mann–Whitney U	1086	0.415
UIBC (µmol/L)	26.30 (21.95–30.75)	30.60 (21.52–36.75)	Mann–Whitney U	1080.5	0.397
AST (U/L)	19.00 (16.00–24.50)	17.00 (14.25–26.50)	Mann–Whitney U	1292	0.669
ALT (U/L)	16.00 (13.00–20.00)	17.00 (13.25–21.75)	Mann–Whitney U	1080.5	0.396
ALP (U/L)	76.00 (58.00–95.00)	77.00 (64.25–95.25)	Mann–Whitney U	1133	0.596
GGT (U/L)	23.00 (17.00–31.00)	33.50 (20.25–48.75)	Mann–Whitney U	868	0.033
CRP (mg/L)	3.20 (1.40–8.20)	4.85 (2.23–13.75)	Mann–Whitney U	1022	0.229
dp-ucMGP (ng/mL)	1.49 (1.26–1.87)	1.24 (1.06–1.46)	Mann–Whitney U	1314.5	0.004

Data are expressed as mean ± standard deviation, median and interquartile range or number of the participants and percentage, depending on the type and distribution of data. ‘Kt/V’—clearance × time/volume (dialysis efficacy indicator), ‘IL-6’—interleukin 6, ‘WBC’—white blood cells, ‘HGB’—hemoglobin, ‘HCT’—hematocrit, ‘PLT’—platelets, ‘LY%’—relative lymphocyte count, ‘MO%’—relative monocyte count, ‘NE%’—relative neutrophil count, ‘LY#’—absolute lymphocyte count, ‘MO#’—absolute monocyte count, ‘NE#’—absolute neutrophil count, ‘RDW’—red blood cells distribution width, ‘MPV’—mean platelets volume, ‘PDW’—platelets distribution width, ‘PCT’—plateletocrit, ‘NLR’—neutrophil–lymphocyte ratio, ‘Na’—sodium concentration in serum, ‘K’—potassium concentration in serum, ‘Ca’—calcium concentration in serum, ‘P’—phosphate concentration, ‘Fe’—iron concentration in serum, ‘TIBC’—total iron binding capacity, ‘UIBC’—unsaturated iron binding capacity, ‘AST’—aspartate aminotransferase, ‘ALT’—alanine aminotransferase, ’ALP’—alkaline phosphatase, ‘GGT’—gamma glutamyl transferase, ‘CRP’—C-reactive protein, ‘dp-ucMGP’—dephosphorylated uncarboxylated Matrix Gla Protein. /: Fisher’s exact test does not return a specific statistic value.

**Table A3:** Spearman correlation analysis between dp-ucMGP, albumin and inflammatory and nutritional markers.

Marker Pair	Spearman Rho (ρ)	*p* Value	N
dp-ucMGP vs. CRP	0.119	0.208	113
dp-ucMGP vs. IL-6	0.093	0.328	113
dp-ucMGP vs. creatinine	0.172	0.069	113
dp-ucMGP vs. NLR	−0.064	0.499	113
dp-ucMGP vs. albumin	0.047	0.618	113
Albumin vs. CRP	−0.354	<0.001	133
Albumin vs. IL-6	−0.389	<0.001	133
Albumin vs. NLR	−0.319	<0.001	133
Albumin vs. creatinine	0.227	0.008	133

Data are expressed as mean ± standard deviation, median and interquartile range or number of the participants and percentage, depending on the type and distribution of data. ‘dp-ucMGP’—dephosphorylated uncarboxylated Matrix Gla Protein, ‘CRP’—C-reactive protein, ‘IL-6’—interleukin 6, ‘NLR’—neutrophil–lymphocyte ratio.

**Table A4:** Comparison of patients included in and excluded from the complete-case analyses.

Variable	Included Patients (N = 113)	Excluded Patients (N = 20)	*p* Value
Age, years	65.00 (56.00–72.00)	65.50 (57.00–71.50)	0.970
Male sex	82/113 (72.6%)	12/20 (60.0%)	0.255
Smoking	35/113 (31.0%)	7/20 (35.0%)	0.721
Hypertension	97/113 (85.8%)	18/20 (90.0%)	1.000
Diabetes mellitus	26/113 (23.0%)	2/20 (10.0%)	0.244
Previous myocardial infarction	14/113 (12.4%)	3/20 (15.0%)	0.721
Previous cerebrovascular event	5/113 (4.4%)	2/20 (10.0%)	0.283
Coronary artery disease	5/113 (4.4%)	0/20 (0.0%)	1.000
Heart failure	7/113 (6.2%)	4/20 (20.0%)	0.062
Arrhythmia	5/113 (4.4%)	2/20 (10.0%)	0.283
Dialysis vintage, months	43.00 (19.00–66.00)	42.50 (18.00–68.50)	0.875
Kt/V	1.23 ± 0.25	1.28 ± 0.36	0.581
IL-6, pg/mL	11.42 (6.41–22.90)	9.18 (4.62–19.19)	0.541
Hemoglobin, g/L	103.23 ± 13.43	109.90 ± 13.22	0.048
NLR	3.40 (2.36–4.45)	3.40 (2.81–3.95)	0.915
Creatinine, µmol/L	915.55 ± 234.25	887.05 ± 196.76	0.567
Total proteins, g/L	65.16 ± 5.23	65.10 ± 5.11	0.962
Albumin, g/L	37.00 (35.00–39.00)	37.50 (34.75–40.25)	0.690
CRP, mg/L	3.90 (1.80–8.70)	2.05 (1.15–4.85)	0.179
All-cause mortality	29/113 (25.7%)	4/20 (20.0%)	0.781
Cardiovascular mortality	20/113 (17.7%)	2/20 (10.0%)	0.526

Data are presented as mean ± standard deviation, median (interquartile range), or N (%), as appropriate. Continuous variables were compared using Welch’s *t*-test or the Mann–Whitney U-test, and categorical variables using the chi-square test or Fisher’s exact test. Kt/V data were available for 103 included and 17 excluded patients; all other variables shown were complete. ‘Kt/V’—clearance × time/volume (dialysis efficacy indicator), ‘IL-6’—interleukin 6, ‘NLR’—neutrophil–lymphocyte ratio, ’CRP’—C-reactive protein.

**Table A5:** Bootstrap internal validation of the combined dp-ucMGP–albumin model.

Endpoint	N	Events	Apparent AUC	Mean Optimism	Optimism-Corrected AUC
All-cause mortality	113	29	0.793	0.009	0.784
Cardiovascular mortality	113	20	0.777	0.017	0.760

Optimism was calculated in each bootstrap resample as the difference between the AUC obtained in the bootstrap sample and the AUC obtained when the model fitted in that resample was applied to the original cohort. The mean optimism was subtracted from the apparent AUC. A total of 1000 valid resamples were analyzed for each endpoint. “AUC”–area under the curve.

**Table A6:** Assessment of the proportional hazards assumption using time-dependent covariates.

Endpoint	Covariate × ln(Time/12)	B	SE	Wald	HR	95% CI	*p* Value
All-cause mortality	Albumin, per 5 g/L × ln(time/12)	0.044	0.154	0.082	1.045	0.772–1.415	0.775
All-cause mortality	dp-ucMGP, per 0.1 ng/mL × ln(time/12)	−0.014	0.041	0.111	0.986	0.910–1.070	0.739
All-cause mortality	Age, per 10 years × ln(time/12)	0.063	0.161	0.150	1.065	0.776–1.461	0.698
Cardiovascular mortality	Albumin, per 5 g/L × ln(time/12)	−0.157	0.201	0.609	0.855	0.577–1.267	0.435
Cardiovascular mortality	dp-ucMGP, per 0.1 ng/mL × ln(time/12)	−0.033	0.043	0.599	0.967	0.888–1.053	0.439
Cardiovascular mortality	Age, per 10 years × ln(time/12)	0.022	0.243	0.008	1.022	0.635–1.645	0.929
Cardiovascular mortality	Smoking, yes vs. no × ln(time/12)	−0.618	0.470	1.725	0.539	0.214–1.355	0.189

Each interaction was tested in a separate extended Cox model while retaining all main covariates of the corresponding primary model. Analysis time was expressed in months and centered at 12 months. The all-cause mortality models included dp-ucMGP, albumin, and age (N = 113; 29 events). The cardiovascular mortality models included dp-ucMGP, albumin, age, and smoking (N = 113; 20 events). ‘HR’- hazard ratio, ‘CI’—confidence interval, ‘dp-ucMGP’—dephosphorylated uncarboxylated matrix Gla protein.

**Table A7:** Sensitivity analyses after exclusion of patients receiving oral vitamin K antagonist therapy.

Endpoint	Analysis	N/Events	dp-ucMGP HR (95% CI); *p*	Albumin HR (95% CI); *p*	Combined-Model AUC (95% CI)
All-cause mortality	Primary	113/29	0.846 (0.768–0.931); 0.001	0.447 (0.311–0.642); <0.001	0.793 (0.708–0.878)
All-cause mortality	Excluding VKA	104/27	0.851 (0.770–0.940); 0.001	0.438 (0.302–0.636); <0.001	0.813 (0.728–0.898)
Cardiovascular mortality	Primary	113/20	0.870 (0.779–0.971); 0.013	0.440 (0.272–0.712); 0.001	0.777 (0.687–0.868)
Cardiovascular mortality	Excluding VKA	104/18	0.866 (0.770–0.974); 0.017	0.420 (0.257–0.686); 0.001	0.797 (0.706–0.888)

Hazard ratios are presented per 0.1 ng/mL increase in dp-ucMGP and per 5 g/L increase in albumin. The all-cause mortality Cox model was adjusted for age, and the cardiovascular mortality model was adjusted for age and smoking. AUC values represent the apparent discriminatory performance of the combined dp-ucMGP–albumin logistic regression model. ‘dp-ucMGP’—dephosphorylated uncarboxylated Matrix Gla Protein, ‘HR’—hazard ratio, ‘CI’—confidence interval, ‘AUC’—area under the curve, ‘VKA’—vitamin K antagonist.

## References

[1] Levey A.S., Coresh J. (2012). Chronic kidney disease. Lancet.

[2] Deng L., Guo S., Liu Y., Zhou Y., Liu Y., Zheng X., Yu X., Shuai P. (2025). Global, regional, and national burden of chronic kidney disease and its underlying etiologies from 1990 to 2021: A systematic analysis for the Global Burden of Disease Study 2021. BMC Public Health.

[3] Bikbov B., Purcell C.A., Levey A.S., Smith M., Abdoli A., Abebe M., Adebayo O.M., Afarideh M., Agarwal S.K., Agudelo-Botero M. (2020). Global, regional, and national burden of chronic kidney disease, 1990–2017: A systematic analysis for the Global Burden of Disease Study 2017. Lancet.

[4] United States Renal Data System (2018). 2018 USRDS Annual Data Report: Epidemiology of Kidney Disease in the United States.

[5] Chen S.I., Chiang C.L., Chao C.T., Chiang C.K., Huang J.W. (2020). Gustatory Function and the Uremic Toxin, Phosphate, Are Modulators of the Risk of Vascular Calcification among Patients with Chronic Kidney Disease: A Pilot Study. Toxins.

[6] Laucyte-Cibulskiene A., Boreikaite E., Aucina G., Gudynaite M., Rudminiene I., Anisko S., Vareikiene L., Gumbys L., Valanciene D., Ryliskyte L. (2018). Usefulness of pretransplant aortic arch calcification evaluation for kidney transplant outcome prediction in one year follow-up. Ren. Fail..

[7] Hutcheson J.D., Goettsch C. (2023). Cardiovascular Calcification Heterogeneity in Chronic Kidney Disease. Circ. Res..

[8] Viegas C., Carreira J., Maia T.M., Macedo A.L., Matos A.P., Neves J., Simes D. (2024). Gla Rich Protein (GRP) Mediates Vascular Smooth Muscle Cell (VSMC) Osteogenic Differentiation, Extracellular Vesicle (EV) Calcification Propensity, and Immunomodulatory Properties. Int. J. Mol. Sci..

[9] Jaminon A.M.G., Dai L., Qureshi A.R., Evenepoel P., Ripsweden J., Soderberg M., Witasp A., Olauson H., Schurgers L.J., Stenvinkel P. (2020). Matrix Gla protein is an independent predictor of both intimal and medial vascular calcification in chronic kidney disease. Sci. Rep..

[10] Malhotra R., Nicholson C.J., Wang D., Bhambhani V., Paniagua S., Slocum C., Sigurslid H.H., Cardenas C.L.L., Li R., Boerboom S.L. (2022). Matrix Gla Protein Levels Are Associated with Arterial Stiffness and Incident Heart Failure with Preserved Ejection Fraction. Arterioscler. Thromb. Vasc. Biol..

[11] Turkmen K., Ozcicek F., Ozcicek A., Akbas E.M., Erdur F.M., Tonbul H.Z. (2014). The relationship between neutrophil-to-lymphocyte ratio and vascular calcification in end-stage renal disease patients. Hemodial. Int..

[12] Stenvinkel P., Ketteler M., Johnson R.J., Lindholm B., Pecoits-Filho R., Riella M., Heimbürger O., Cederholm T., Girndt M. (2005). IL-10, IL-6, and TNF-α: Central factors in the altered cytokine network of uremia—The good, the bad, and the ugly. Kidney Int..

[13] Graterol Torres F., Molina M., Soler-Majoral J., Romero-González G., Rodríguez Chitiva N., Troya-Saborido M., Rullan G.S., Burgos E., Martínez J.P., Jou M.U. (2022). Evolving Concepts on Inflammatory Biomarkers and Malnutrition in Chronic Kidney Disease. Nutrients.

[14] Ignjatovic A.M., Cvetkovic T.P., Pavlovic R.M., Djordjevic V.M., Milosevic Z.G., Djordjevic V.B., Pavlović D.D., Stojanović I.R., Bogdanović D. (2013). Endothelial dysfunction, inflammation and malnutrition as predictors of mortality in dialysis patients: Multimarker approach. Int. Urol. Nephrol..

[15] Faintuch J., Morais A.A.C., Silva M.A.T., Vidigal E.J., Costa R.A., Lyrio D.C., Trindade C.R., Pitanga K.K. (2006). Nutritional Profile and Inflammatory Status of Hemodialysis Patients. Ren. Fail..

[16] Sabatino A., Regolisti G., Karupaiah T., Sahathevan S., Singh B.S., Khor B.H., Salhab N., Karavetian M., Cupisti A., Fiaccadori E. (2017). Protein-energy wasting and nutritional supplementation in patients with end-stage renal disease on hemodialysis. Clin. Nutr..

[17] Keller U. (2019). Nutritional Laboratory Markers in Malnutrition. J. Clin. Med..

[18] Roumeliotis S., Roumeliotis A., Stamou A., Leivaditis K., Kantartzi K., Panagoutsos S., Liakopoulos V. (2020). The Association of dp-ucMGP with Cardiovascular Morbidity and Decreased Renal Function in Diabetic Chronic Kidney Disease. Int. J. Mol. Sci..

[19] Willeit K., Santer P., Tschiderer L., Pechlaner R., Vermeer C., Willeit J., Kiechl S. (2022). Association of desphospho-uncarboxylated matrix gla protein with incident cardiovascular disease and all-cause mortality: Results from the prospective Bruneck Study. Atherosclerosis.

[20] Fine A. (2002). Relevance of C-reactive protein levels in peritoneal dialysis patients. Kidney Int..

[21] Sproston N.R., Ashworth J.J. (2018). Role of C-Reactive Protein at Sites of Inflammation and Infection. Front. Immunol..

[22] Roch P.J., Ecker C., Jäckle K., Meier M.P., Reinhold M., Klockner F.S., Lehmann W., Weiser L. (2024). Interleukin-6 as a critical inflammatory marker for early diagnosis of surgical site infection after spine surgery. Infection.

[23] Sahib A., Choudhury C., Wani I.A., Wani M.M. (2024). Evaluation of Inflammatory Status in Chronic Kidney Disease Patients and a Comparison Between Hemodialysis and Peritoneal Dialysis Patients. Cureus.

[24] Jofré R., Rodriguez-Benitez P., López-Gómez J.M., Pérez-Garcia R. (2006). Inflammatory Syndrome in Patients on Hemodialysis. J. Am. Soc. Nephrol..

[25] Ebert T., Neytchev O., Witasp A., Kublickiene K., Stenvinkel P., Shiels P.G. (2021). Inflammation and Oxidative Stress in Chronic Kidney Disease and Dialysis Patients. Antioxid. Redox Signal..

[26] Elangovan D., Krishnamoorthy S., Thiyagarajan S., Silambanan S. (2024). Association of NLR, MLR, PLR, SII, and SIRI with the stages of chronic kidney disease—A cross-sectional study. Int. J. Med. Biochem..

[27] Yazıcı R., Güney İ. (2023). Prognostic value of inflammatory markers for mortality in hemodialysis patients: A retrospective study with over 3-year follow-up. J. Health Sci. Med..

[28] Zhu Q., Dai L. (2025). Prognostic implications of systemic immune-inflammation index and systemic inflammation response index in hemodialysis patients. BMC Nephrol..

[29] Seo I.H., Lee Y.J. (2022). Usefulness of Complete Blood Count (CBC) to Assess Cardiovascular and Metabolic Diseases in Clinical Settings: A Comprehensive Literature Review. Biomedicines.

[30] Mayer O., Seidlerová J., Bruthans J., Filipovský J., Timoracká K., Vaněk J., Černá L., Wohlfahrt P., Cífková R., Theuwissen E. (2014). Desphospho-uncarboxylated matrix Gla-protein is associated with mortality risk in patients with chronic stable vascular disease. Atherosclerosis.

[31] Xu Q., Guo H., Cao S., Zhou Q., Chen J., Su M., Chen S., Jiang S., Shi X., Wen Y. (2019). Associations of vitamin K status with mortality and cardiovascular events in peritoneal dialysis patients. Int. Urol. Nephrol..

[32] Schlieper G., Westenfeld R., Krüger T., Cranenburg E.C., Magdeleyns E.J., Brandenburg V.M., Djuric Z., Damjanovic T., Ketteler M., Vermeer C. (2011). Circulating Nonphosphorylated Carboxylated Matrix Gla Protein Predicts Survival in ESRD. J. Am. Soc. Nephrol..

[33] Schurgers L.J., Barreto D.V., Barreto F.C., Liabeuf S., Renard C., Magdeleyns E.J., Vermeer C., Choukroun G., Massy Z.A. (2010). The Circulating Inactive Form of Matrix Gla Protein Is a Surrogate Marker for Vascular Calcification in Chronic Kidney Disease: A Preliminary Report. Clin. J. Am. Soc. Nephrol. CJASN.

[34] Mulder M.M.G., Schellens J., Sels J.W.E.M., van Rosmalen F., Hulshof A.M., de Vries F., Segers R., Mihl C., van Mook W.N.K.A., Bast A. (2023). Higher levels of circulating desphospho-uncarboxylated matrix Gla protein over time are associated with worse survival: The prospective Maastricht Intensive Care COVID cohort. J. Intensive Care.

[35] Palamar M., Grosu I.D., Schiller A., Petrica L., Bodea M., Sircuta A., Gruescu E., Matei O.D., Tanasescu M.D., Golet I. (2025). Vitamin K-Dependent Proteins as Predictors of Valvular Calcifications and Mortality in Hemodialysis Patients. Biomedicines.

[36] Soeters P.B., Wolfe R.R., Shenkin A. (2019). Hypoalbuminemia: Pathogenesis and Clinical Significance. J. Parenter. Enter. Nutr..

[37] Aoun M., Makki M., Azar H., Matta H., Chelala D.N. (2017). High Dephosphorylated-Uncarboxylated MGP in Hemodialysis patients: Risk factors and response to vitamin K_2_, A pre-post intervention clinical trial. BMC Nephrol..

[38] Zoanni B., Brioschi M., Mallia A., Gianazza E., Eligini S., Carini M., Aldini G., Banfi C. (2023). Novel insights about albumin in cardiovascular diseases: Focus on heart failure. Mass Spectrom. Rev..

[39] Chen X., Wang G., Yin X., Liu W., Huang H., Li D. (2025). Inflammatory and nutritional indices for overall survival in Hemodialysis patients: A multicenter cohort study. BMC Nephrol..

